# Supplementary material for: RNA demethylase FTO participates in malignant progression of gastric cancer by regulating SP1-AURKB-ATM pathway
Source: Commun Biol. 2024 Jul 2;7:800. doi: 10.1038/s42003-024-06477-y (PMC11220007; doi:10.1038/s42003-024-06477-y)
Supplement: Supplementary file 2 — Supplementary Information [file 42003_2024_6477_MOESM2_ESM.pdf]

Supplementary Figure1

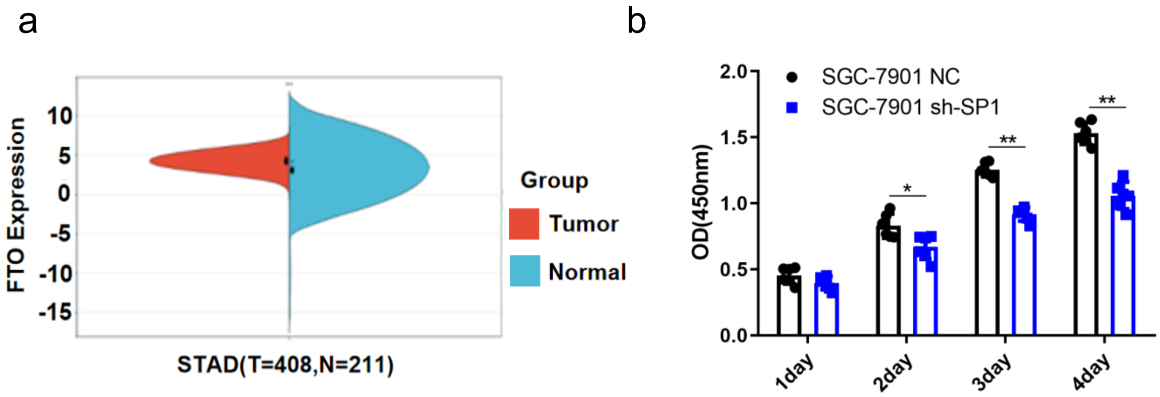

Supplementary Figure1.
FTO is highly expressed in gastric cancer. (a) FTO expression levels in STAD (stomach adenocarcinoma) and normal tissues from the TCGA database. (b) The cell viability of SGC7901 cells were tested by CCK-8. $*p < 0.05$, $**p < 0.01$.

Supplementary Figure2

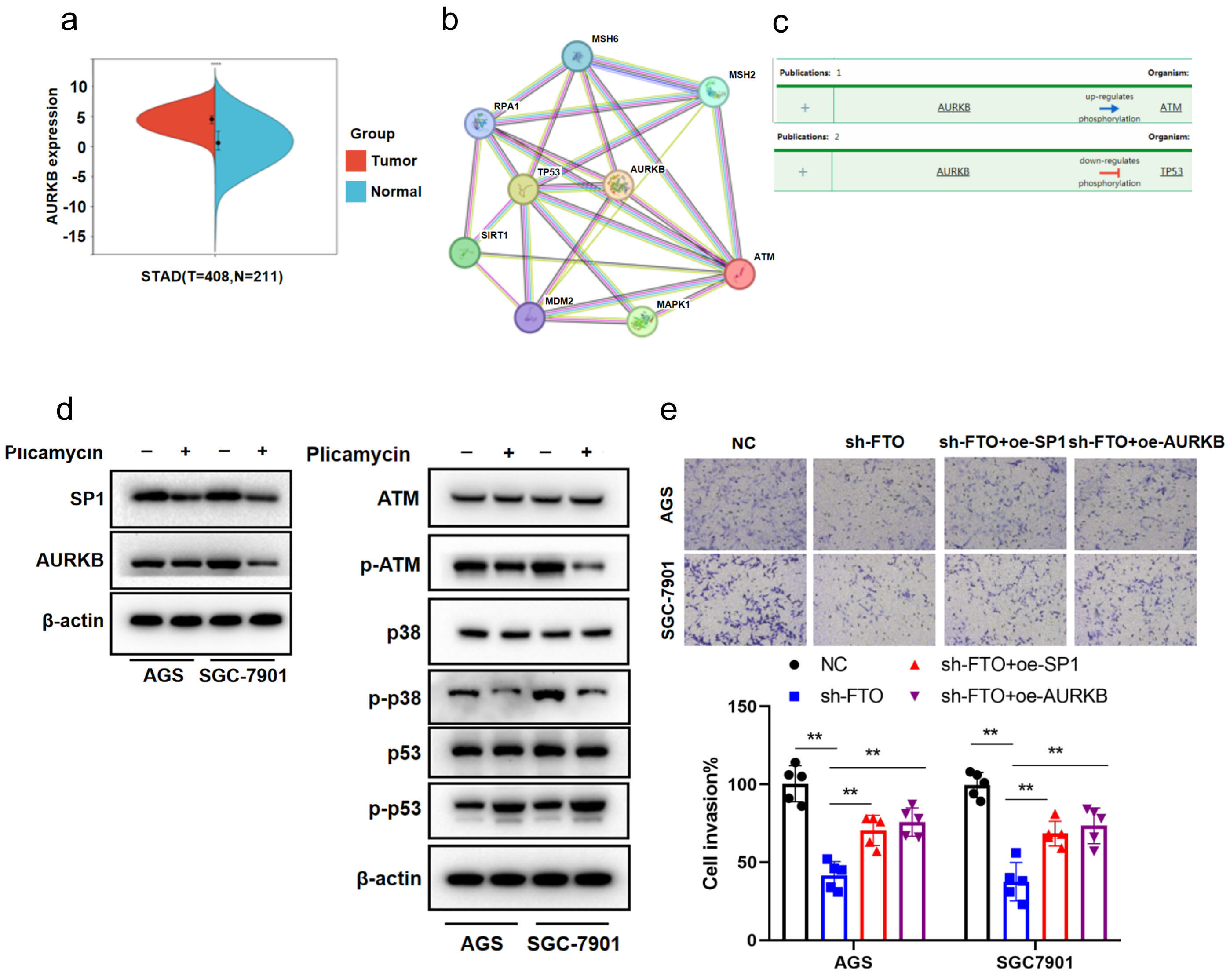

Supplementary Figure2.

FTO promotes gastric cancer progression through the SP1-ATM signal axis. (a) AURKB expression levels in STAD (stomach adenocarcinoma) and normal tissues from the TCGA database. (b) The protein-to-protein interaction network was performed with STRING website. (c) The downstream analysis of AURKB was performed with SIGNOR website. (d) GC cells were treated with SP1 inhibitor Plicamycin to confirm the downstream pathway of SP1. (e) Invasion ability of GC were tested through Transwell assay. *$p < 0.05$, **$p < 0.01$.

Supplementary Figure3

a

Chr	start	end	strand	gene_id	gene_name	transcript	GO	Description	fc	log2(fc)	pval	qval	regulation	significant
chr12	20815674	21090245	+	ENSG00002	SLCO1B3-S	ENST00000	GO:0005515(protei	SLCO1B3-SLCO1B7 r	2.30	1.20	0.03	0.28	up	yes
chr7	5592816	5606655	+	ENSG00000	FSCN1	ENST00000	GO:0001725(stress	fascin actin-bund	2.22	1.15	0.04	0.32	up	yes
chr11	102770502	102780628	-	ENSG00000	MMP10	ENST00000	GO:0004222(metall	matrix metallopep	2.06	1.05	0.03	0.27	up	yes
chr12	55757275	55827546	-	ENSG00002	ENSG00000	ENST00000	GO:0005515(protei	novel protein	3.39	1.76	0.03	0.28	up	yes
chr12	53380176	53416446	+	ENSG00000	SP1	ENST00000	GO:0000785(chroma	Sp1 transcription	2.50	1.32	0.02	0.17	up	yes
chr9	6215786	6257983	+	ENSG00000	IL33	ENST00000	GO:0000122(negati	interleukin 33 [S	5.18	2.37	0.01	0.09	up	yes
chr1	153039732	153041931	-	ENSG00000	SPRR2D	ENST00000	GO:0001533(cornif	small proline ric	2.12	1.08	0.04	0.32	up	yes
chr9	97198303	97199511	-	ENSG00000	ZNF322P1	ENST00000	NA	zinc finger prote	2.17	1.12	0.03	0.26	up	yes
chr1	94148988	94275068	-	ENSG00000	ARHGAP29	ENST00000	GO:0005096(GTPase	Rho GTPase activa	7.64	2.93	0.00	0.00	up	yes
chr15	51447711	51622833	-	ENSG00000	DMXL2	ENST00000	GO:0005515(protei	Dmx like 2 [Sourc	2.20	1.14	0.05	0.35	up	yes
chr19	54200809	54249003	+	ENSG00000	RPS9	ENST00000	GO:0002181(cytopl	ribosomal protein	2.00	1.00	0.05	0.36	up	yes
chr4	108620569	108630412	+	ENSG00000	RPL34	ENST00000	GO:0002181(cytopl	ribosomal protein	2.05	1.03	0.04	0.34	up	yes
chr19	18153163	18170532	+	ENSG00000	PIK3R2	ENST00000	GO:0001678(cellul	phosphoinositide-	76.71	6.26	0.00	0.04	up	yes
chr1	35713877	35718894	-	ENSG00000	C1orf216	ENST00000	GO:0005515(protei	chromosome 1 open	22.13	4.47	0.00	0.01	up	yes
chr1	160997957	161038962	-	ENSG00002	ENSG00000	ENST00000	NA	novel transcript,	18.26	4.19	0.03	0.29	up	yes
chr19	23255053	23257939	-	ENSG00002	IPO5P1	ENST00000	NA	importin 5 pseudo	18.11	4.18	0.01	0.15	up	yes
chr1	149390623	149556361	+	ENSG00002	ENSG00000	ENST00000	NA	novel protein, id	14.27	3.84	0.03	0.28	up	yes
chr11	57712582	57818431	+	ENSG00002	ENSG00000	ENST00000	GO:0016020(membra	TMX2-CTNND1 readt	13.90	3.80	0.03	0.24	up	yes
chr2	233865496	233867359	-	ENSG00002	ENSG00000	ENST00000	NA	novel transcript	12.68	3.66	0.05	0.35	up	yes
chr6	41736711	41754109	-	ENSG00000	PGC	ENST00000	GO:0002803(positi	progastricsin [So	12.32	3.62	0.00	0.07	up	yes
chr7	102537918	102572653	-	ENSG00000	POLR2J3	ENST00000	GO:0000428(DNA-di	RNA polymerase II	0.02	-5.64	0.00	0.00	down	yes
chr17	22090743	22205156	+	ENSG00002	UBBP4	ENST00000	NA	ubiquitin B pseud	0.05	-4.22	0.00	0.00	down	yes
chr1	226940286	226987544	+	ENSG00000	COQ8A	ENST00000	GO:0000166(nucleo	coenzyme Q8A [Sou	0.09	-3.53	0.00	0.00	down	yes
chr20	23685640	23689038	-	ENSG00000	CST4	ENST00000	GO:0001580(detect	cystatin S [Sourc	0.11	-3.24	0.01	0.13	down	yes
chr19	44914247	44919349	+	ENSG00000	APOC1	ENST00000	GO:0004859(phosph	apolipoprotein C1	0.18	-2.49	0.00	0.02	down	yes
chr18	39334872	39335672	-	ENSG00000	RPL7AP66	ENST00000	NA	ribosomal protein	0.11	-3.14	0.00	0.01	down	yes
X	1403139	1453762	-	ENSG00000	ASMTL	ENST00000	GO:0003674(molecu	acetylserotonin O	0.16	-2.65	0.00	0.02	down	yes
chr19	51345169	51366388	-	ENSG00000	ETFB	ENST00000	GO:0005515(protei	electron transfer	0.13	-2.97	0.00	0.01	down	yes
X	49829260	49834264	+	ENSG00000	PAGE4	ENST00000	GO:0003677(DNA bi	PAGE family membe	0.15	-2.76	0.00	0.00	down	yes
chr9	129175552	129178261	-	ENSG00000	IER5L	ENST00000	NA	immediate early r	0.17	-2.52	0.00	0.00	down	yes
chr11	94021354	94114208	+	ENSG00000	HEPHL1	ENST00000	GO:0004322(ferrox	hephaestin like 1	0.13	-2.93	0.00	0.01	down	yes
chr1	16514122	16514285	-	ENSG00002	RNU1-1	ENST00000	GO:0000395(mRNA 5	RNA, U1 small nuc	0.13	-2.90	0.00	0.01	down	yes
chr13	52024691	52033600	+	ENSG00002	UTP14C	ENST00000	GO:0005515(protei	UTP14C small subu	0.13	-2.89	0.00	0.01	down	yes
chr2	107826892	107892544	+	ENSG00000	RGPD4	ENST00000	GO:0005096(GTPase	RANBP2 like and G	0.14	-2.88	0.00	0.02	down	yes
chr2	178829757	179050137	-	ENSG00000	CCDC141	ENST00000	GO:0005515(protei	coiled-coil domai	0.14	-2.88	0.00	0.01	down	yes
chr12	7812512	7891148	+	ENSG00000	SLC2A14	ENST00000	GO:0005355(glucos	solute carrier fa	0.14	-2.86	0.00	0.02	down	yes
chr8	134792020	134798272	-	ENSG00002	MIR30DHG	ENST00000	NA	MIR30D and MIR30B	0.14	-2.83	0.00	0.01	down	yes
chr1	94148988	94275068	-	ENSG00000	ARHGAP29	ENST00000	GO:0005096(GTPase	Rho GTPase activa	0.15	-2.76	0.00	0.00	down	yes
chr4	119212587	119295518	+	ENSG00000	USP53	ENST00000	GO:0001508(action	ubiquitin specifi	0.15	-2.72	0.00	0.00	down	yes
chr5	108747841	109196841	+	ENSG00000	FER	ENST00000	GO:0000166(nucleo	FER tyrosine kina	0.15	-2.70	0.00	0.00	down	yes

b

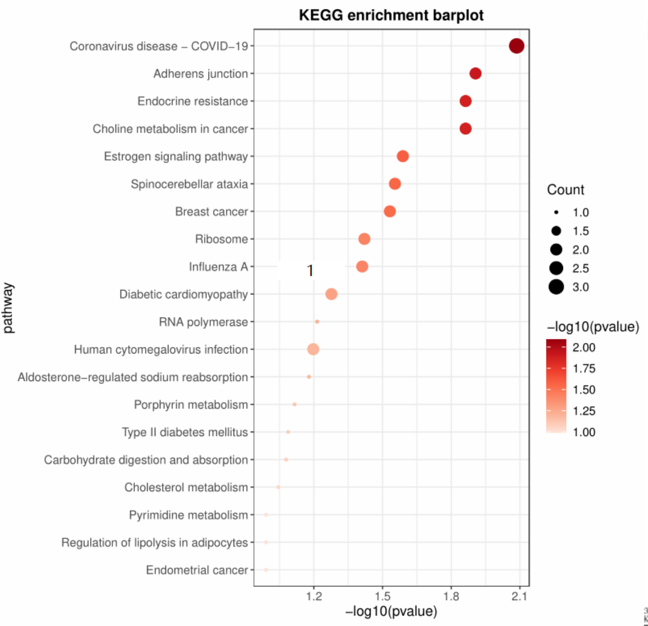

c

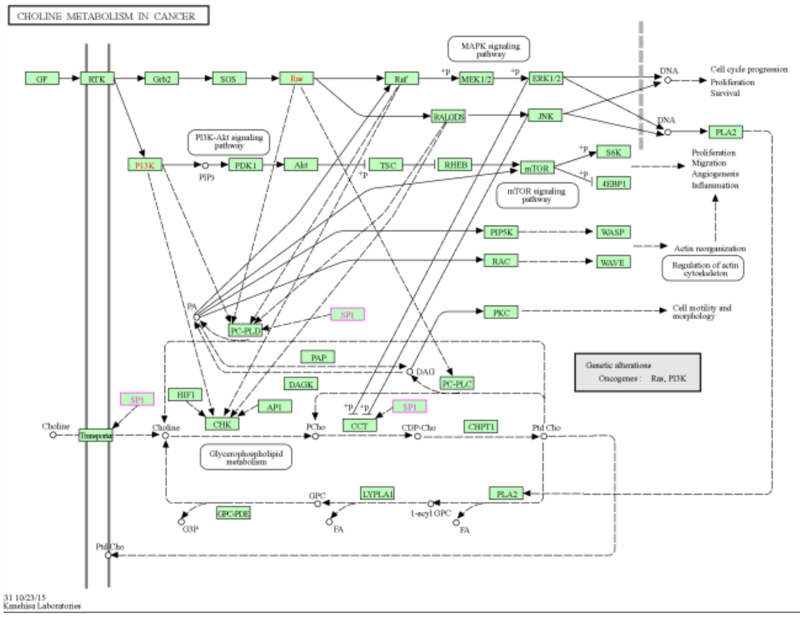

Supplementary Figure3.

FTO regulates SP1 expression. (a) List of differential genes by methylation sequencing (pIKO.1 vs. shFTO); (b) KEGG analysis of differential genes; (c) KEGG analysis of SP1.

Supplementary Figure4

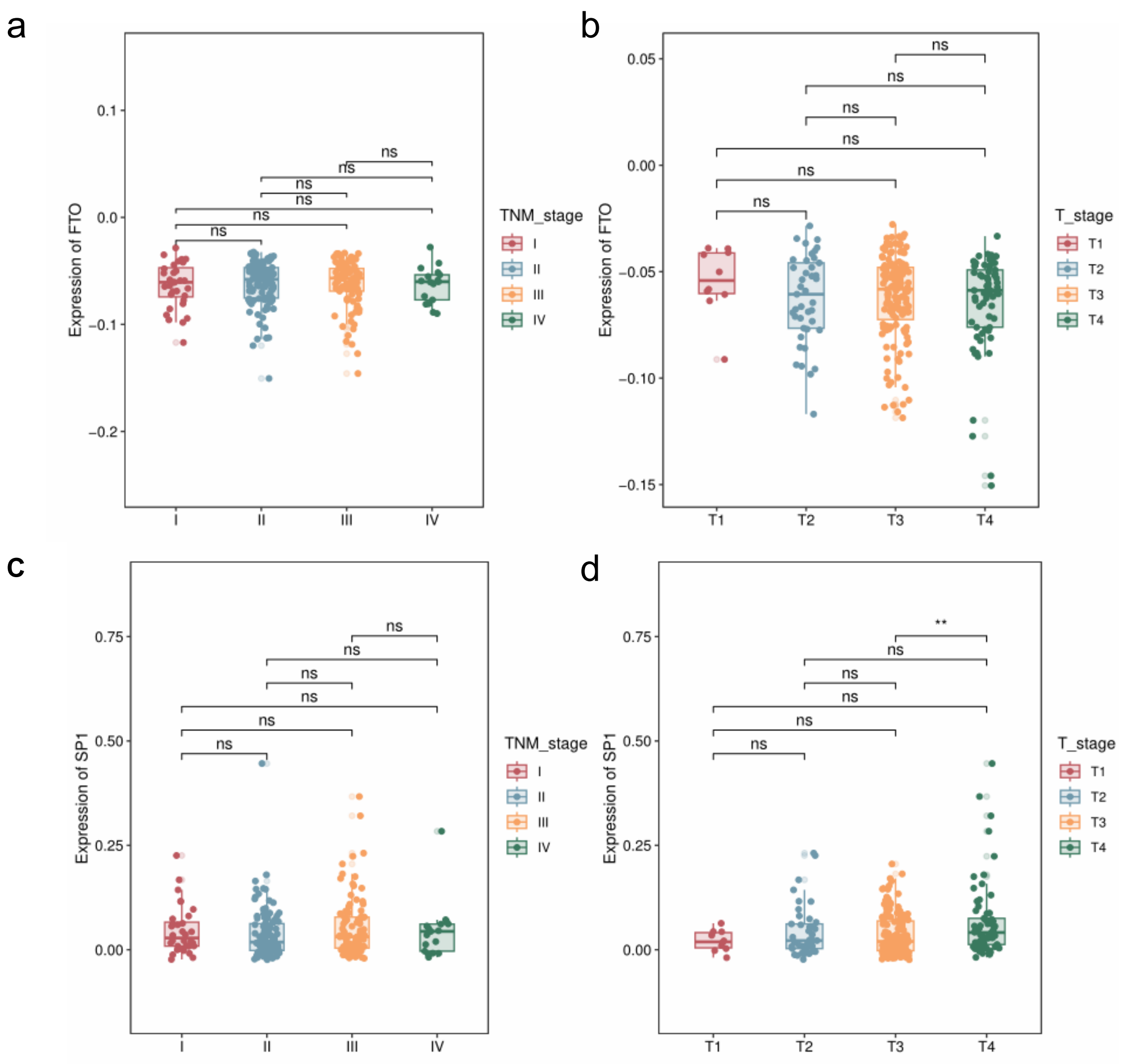

Supplementary Figure4

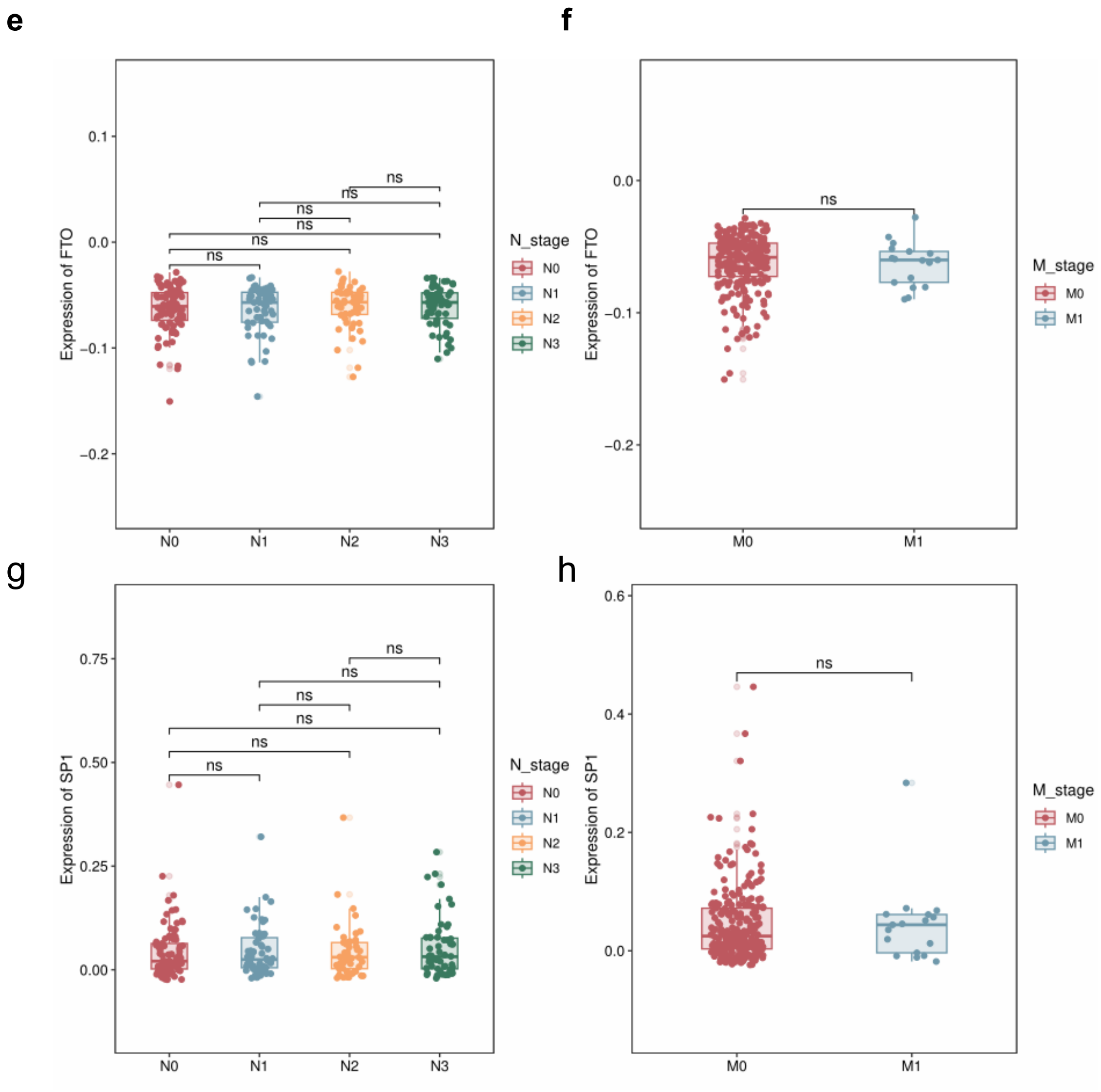

Supplementary Figure4.

Expression of FTO and SP1 in different stages of gastric cance. (a) No statistical significance was observed in correlation between the FTO expression and TNM stage; (b) There were no associations between FTO expression and T stage; (c) No statistical significance was observed in correlation between the SP1 expression and TNM stage; (d) There were no associations between SP1 expression and T stage; (e) There was no significant correlation between expression of FTO and N stage classification; (f) FTO expression was not significantly associated with M stage; (g) There was no significant correlation between expression of SP1 and N stage classification; (h) SP1 expression was not significantly associated with M stage.

Supplementary Figure5

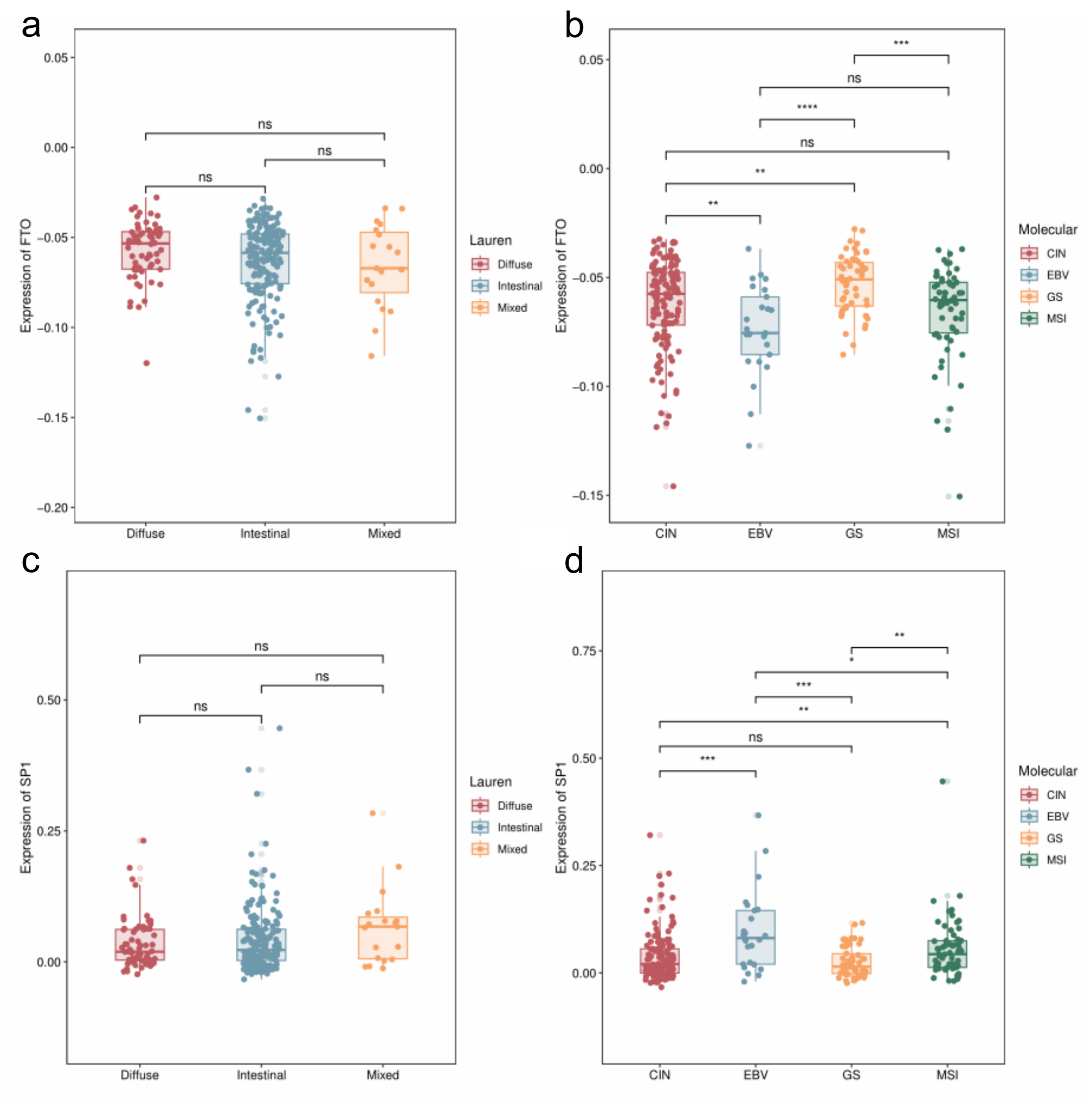

Supplementary Figure5.

Expression of FTO and SP1 in different types of gastric cance. (a) No significant association of FTO expression with Lauren classification; (b) There was no obvious correlation between the expression of FTO and the molecular classification of GC; (c) No significant association of SP1 expression with Lauren classification; (d) There was no obvious correlation between the expression of FTO and the molecular classification of GC.

Supplementary Figure6

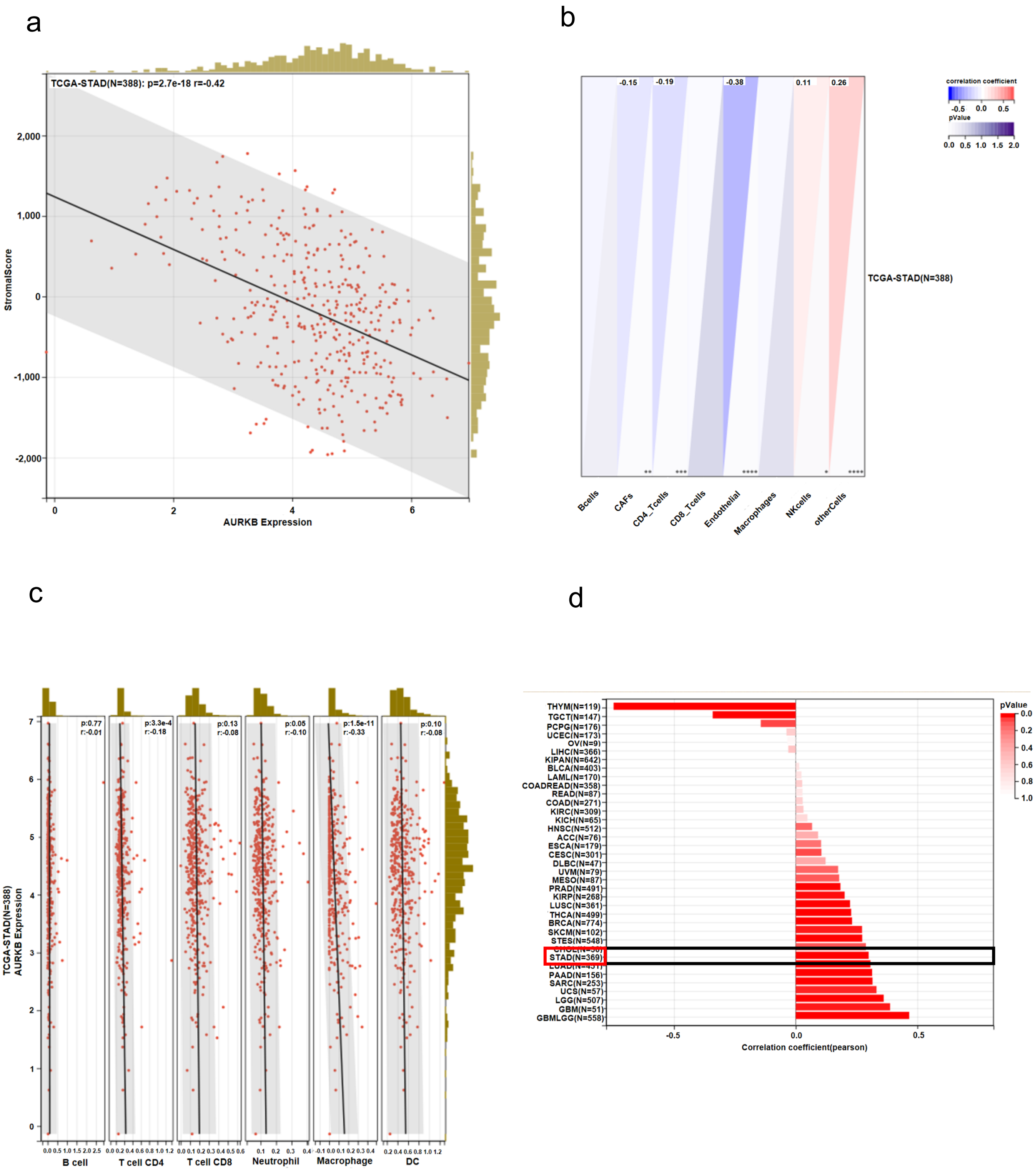

Supplementary Figure6.

Relationship between AURKB expression and microenvironment of gastric cancer. (a) StromalScore of AURKB expression in STAD. (b-c) Differential analysis of immune cell components in high AURKB expression GC tumors. (d) The correlation between AURKB expression and tumor stemness index.

Supplementary Figure7

Figure 1c FTO

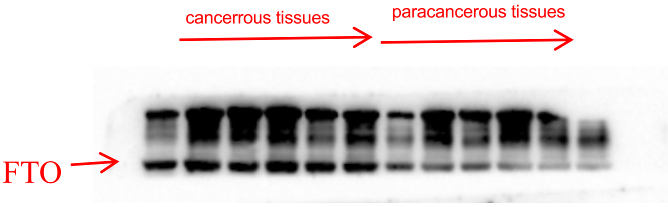

Figure 1c β-actin

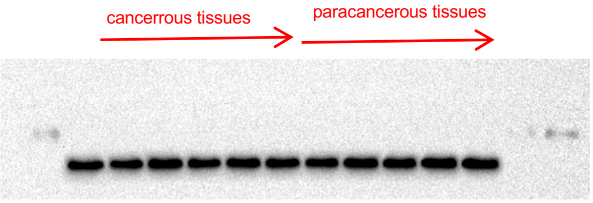

Figure 1c FTO

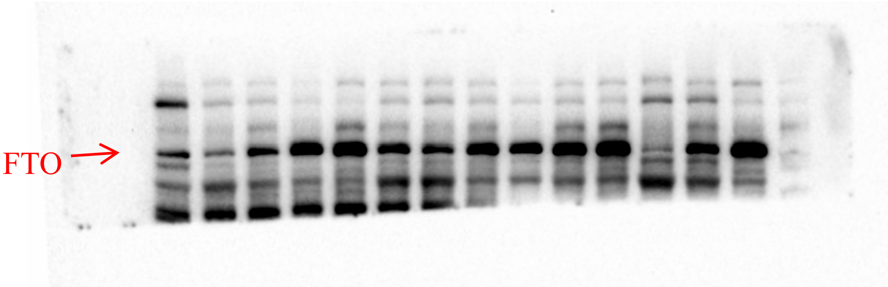

Figure 1c β-actin

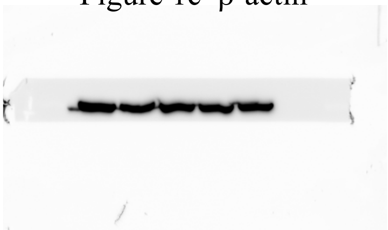

Figure 2b FTO

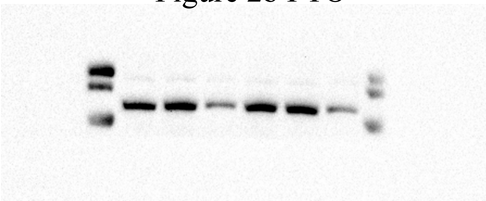

Figure 2b β-actin

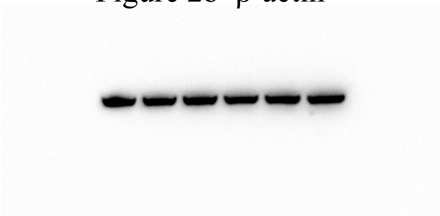

Figure 3g SP1

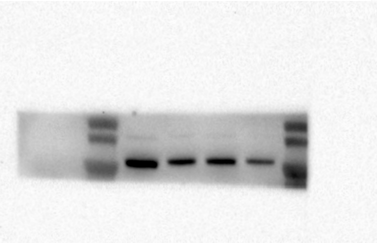

Figure 3g β-actin

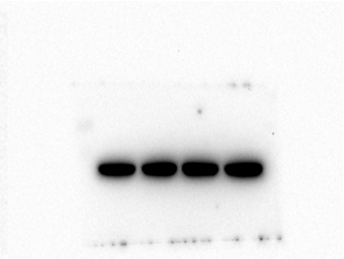

Figure 3j SP1

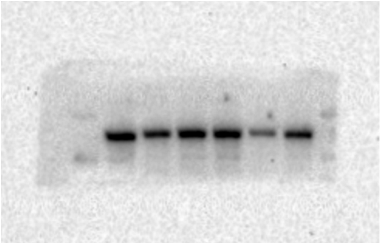

Figure 3j β-actin

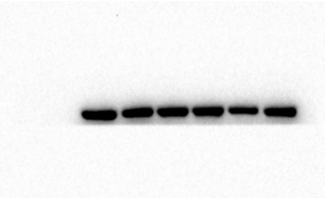

Figure 4d SP1

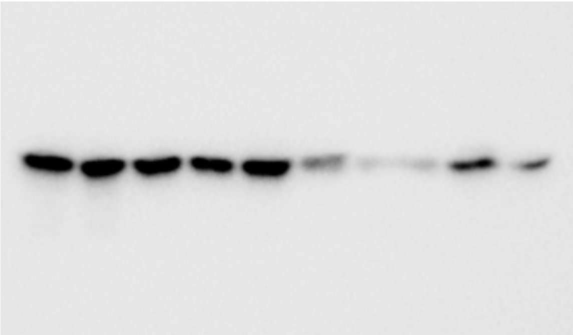

Figure 4d β-actin

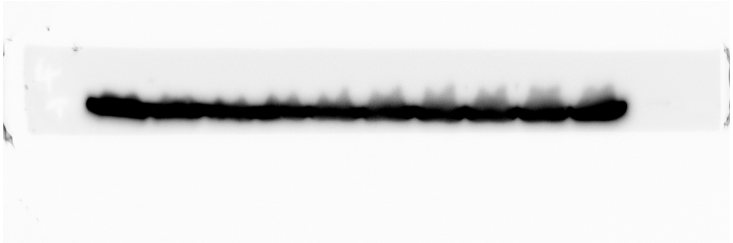

Supplementary Figure7

Figure 6c SP1

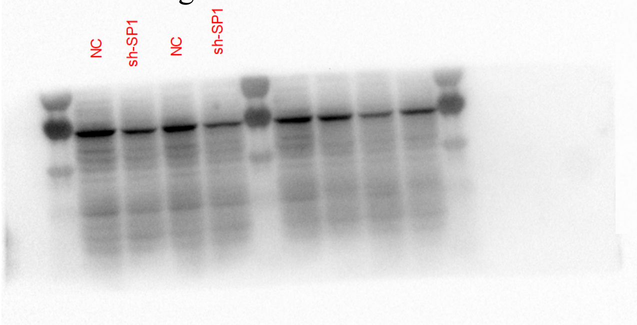

Figure 6c AURKB

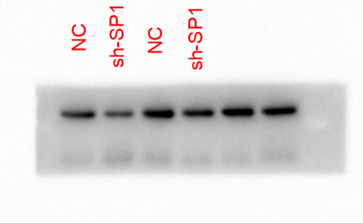

Figure 6c β-actin

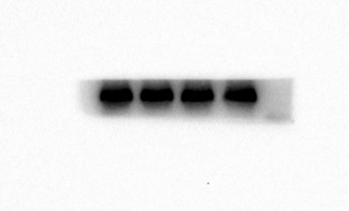

Figure 6e AURKB

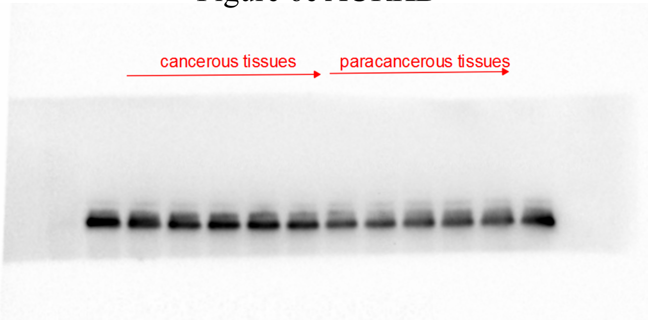

Figure 6e β-actin

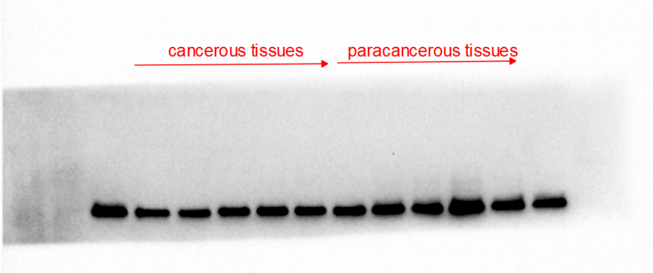

Figure 6h FTO

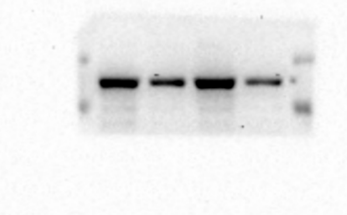

Figure 6h β-actin

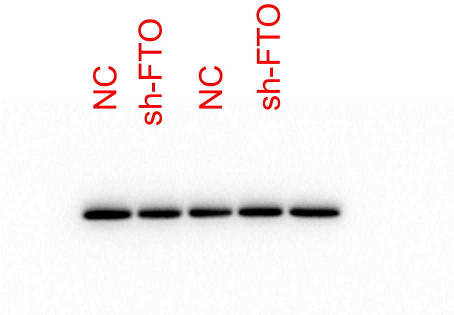

Figure 6j ATM (AURKB-knockdown)

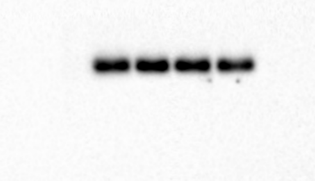

Figure 6j p38 (AURKB-knockdown)

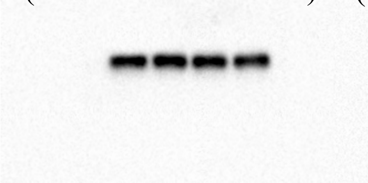

Figure 6j p53 (AURKB-knockdown)

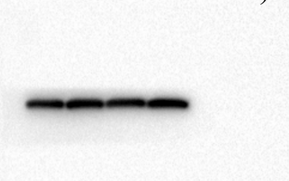

Figure 6j p-ATM (AURKB-knockdown)

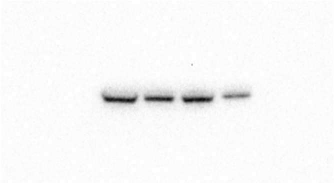

Figure 6j p-p38 (AURKB-knockdown)

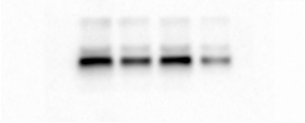

Figure 6j p-p53 (AURKB-knockdown)

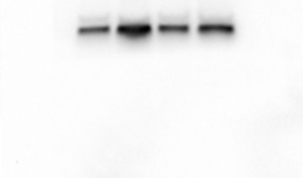

Figure 6j β-actin (AURKB-knockdown)

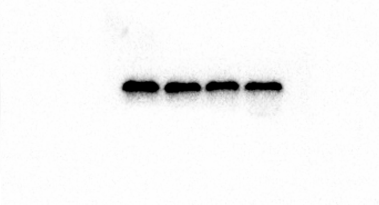

Supplementary Figure7

Figure 6j ATM (SP1-knockdown)

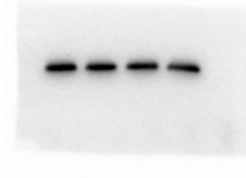

Figure 6j p38 (SP1-knockdown)

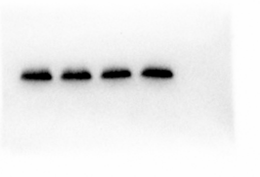

Figure 6j p53 (SP1-knockdown)

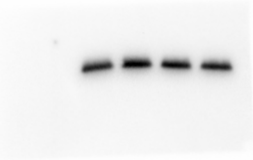

Figure 6j p-ATM (SP1-knockdown)

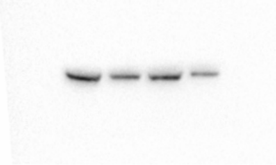

Figure 6j p-p38 (SP1-knockdown)

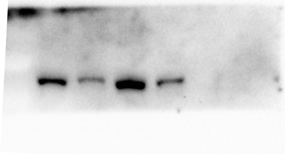

Figure 6j p-p53 (SP1-knockdown)

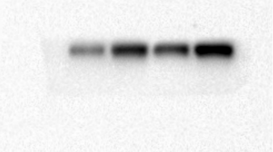

Figure 6j β-actin (SP1-knockdown)

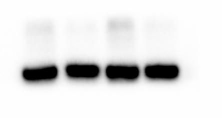

Figure 7d ATM (AGS cell)

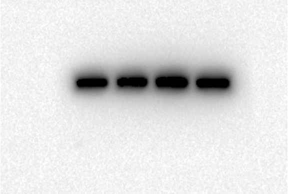

Figure 7d p38 (AGS cell)

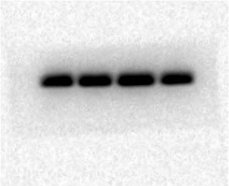

Figure 7d p53 (AGS cell)

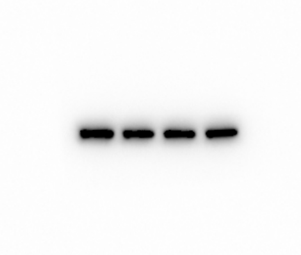

Figure 7d p-ATM (AGS cell)

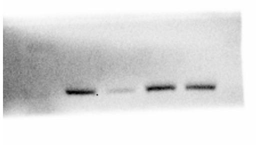

Figure 7d p-p38 (AGS cell)

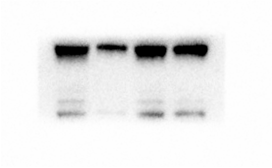

Figure 7d p-p5 (AGS cell)

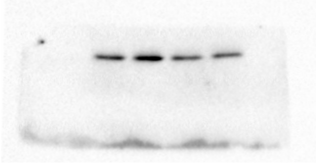

Figure 7d β-actin (AGS cell)

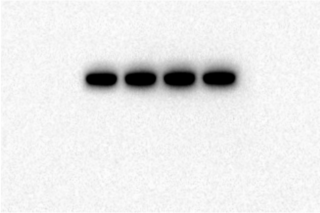

Figure 7d ATM (SGC7901 cell)

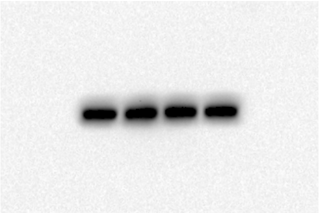

Figure 7d p38 (SGC7901 cell)

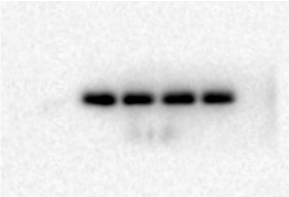

Figure 7d p53 (SGC7901 cell)

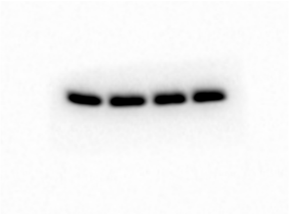

Figure 7d p-ATM (SGC7901 cell)

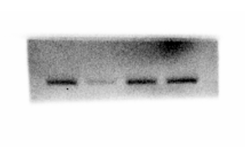

Figure 7d p-p38 (SGC7901 cell)

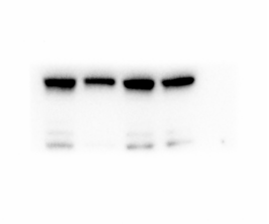

Figure 7d p-p53 (SGC7901 cell)

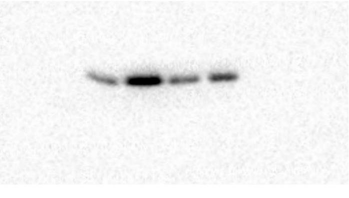

Figure 7d β-actin (SGC7901 cell)

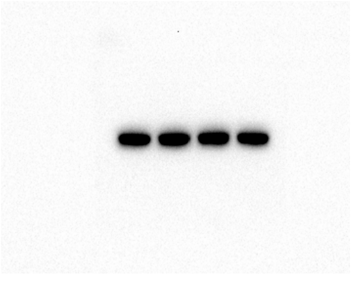

Supplementary Figure7

Supplementary Figure2d SP1

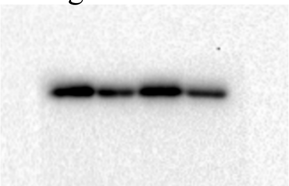

Supplementary Figure2d ATM

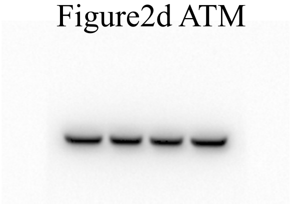

Supplementary Figure2d p38

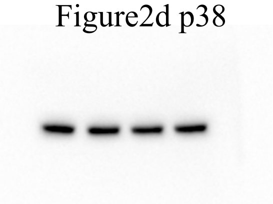

Supplementary Figure2d p53

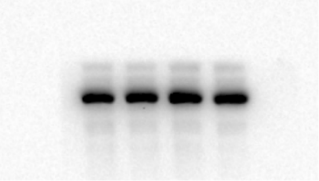

Supplementary Figure2d AURKB

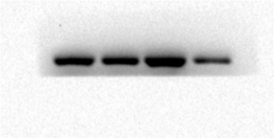

Supplementary Figure2d p-ATM

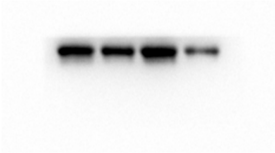

Supplementary Figure2d p-p38

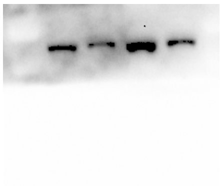

Supplementary Figure2d p-p53

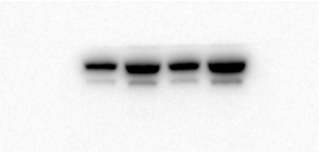

Supplementary Figure2d β-actin

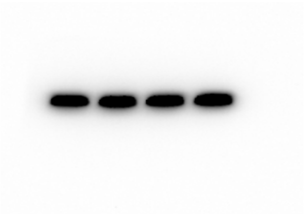

Supplementary Figure2d β-actin

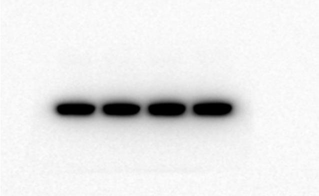

Supplementary Figure7.
Unprocessed scans of blots in article

Supplementary table1

The statistics of GC cases

Number	Gender	Age	Nerve invasion	Tumor differentiation	Tumor size(cm)	LNM
1	F	82	P	L	4	2
2	M	56	N	M	1.5	0
3	M	61	P	L	6	26
4	M	70	N	L	6.5	1
5	M	63	N	L	5	0
6	M	65	P	L	1	7
7	M	63	P	M	6.5	21
8	M	68	P	L	5	0
9	M	67	P	M	6	3
10	F	65	P	L	5	0
11	M	66	P	M	3	6
12	F	47	P	L	2	10
13	M	56	N	L	3.5	0
14	M	44	P	L	6	6
15	F	55	P	M	8	1
16	M	65	P	M	1.5	0
17	M	53	N	L	3	1
18	M	75	N	M	3	0
19	M	65	P	L	6	25
20	F	58	P	L	7	5

Supplementary table2

PCR primers and shRNA sequences

Primers	Sequences
FTO Forward primer	TTGCATGGATGAGCCAGCTT
FTO Reverse primer	TCTCCAACCCTGTTGCACAT
SP1 Forward primer	CCACCATGAGCGACCAAGA
SP1 Reverse primer	GGTACTGCTGCCACTCTGTT
AURKB Forward primer	GGAGAGTAGCAGTGCCTTGG
AURKB Reverse primer	GACAAGTGCAGATGGGGTGA
YTHDF2 Forward primer	GTGTCAGGGACAAAAGCCTCC
YTHDF2 Reverse primer	GCCGACATGGCTCTCAGAT
sequence of FTO shRNA	TCACCAAGGAGACTGCTATTT
sequence of SP1 shRNA	GCTGGTGGTGATGGAATACAT
sequence of YTHDF2 shRNA	GATGGATTAAACGATGATGAT
